# Relationship between impulse and kinetic variables during jumping and landing in volleyball players

**DOI:** 10.1186/s12891-023-06757-4

**Published:** 2023-07-29

**Authors:** Razieh Yousefian Molla, Ali Fatahi, Davood Khezri, Halil Ibrahim Ceylan, Hadi Nobari

**Affiliations:** 1 Department of Physical Education and Sport Sciences, Islamic Azad University of Karaj Branch, Karaj, Iran; 2 Department of Sports Biomechanics, Central Tehran branch, Islamic Azad University, Tehran, Iran; 3 Department of Sport Biomechanics and Technology, Sport Sciences Research Institute, Tehran, 1587958711 Iran; 4 Physical Education and Sports Teaching Department, Kazim Karabekir Faculty of Education, Ataturk University, Erzurum, Turkey; 5 Faculty of Sport Sciences, University of Extremadura, Cáceres, 10003 Spain; 6 Department of Exercise Physiology, Faculty of Educational Sciences and Psychology, University of Mohaghegh Ardabili, Ardabil, 56199-11367 Iran

**Keywords:** Concentric, Kinetic, rate of force development, Peak power, Performance, Sport teams

## Abstract

**Background:**

This study examined the relationships between impulse and kinetic variables during jumping and landing in elite young male volleyball players.

**Methodology:**

Eighteen players were recruited and asked to jump on a force plate, which allowed for the direct extraction of jump and landing kinetic data. The data was then analysed using stepwise regression to explore the relationship between landing impulse and various kinetic variables.

**Results:**

Our findings revealed a significant positive relationship between the peak rate of force development concentric (PRFD _CON_) and impulse at landing (β = 0.537, p = 0.02). In a secondary analysis, we found that PRFD _CON_ (β = 0.497, p = 0.01) and time to peak power concentric (TPPC) (β = 0.424, p = 0.04) were also positively correlated with landing impulse. Importantly, PRFD _CON_ and TPPC were the variables that had the most muscular predictive power for impulse at landing.

**Conclusion:**

These findings offer crucial insights into the biomechanics of jumping and landing in elite young male volleyball players, informing the development of more effective training programs. Our study identifies PRFD _CON_ and TPPC as critical factors for improving landing impulse, emphasizing the need to consider multiple kinetic variables when designing training programs for explosive skills. These insights can help optimize performance and reduce the risk of injury in elite young male volleyball players.

## Introduction

Jumping, followed by landing, is one of the most critical performance indicators for success in many sports [1, 2]. Identifying risk factors is essential to prevent injuries [3]. Concerning jump and landing skills, kinetic parameters play a significant role in the incidence of sports injuries and performance optimization [2, 4]. Jumping, which involves a cycle of tension-contraction, takes place in distinct eccentric, amortization, and concentric stages before the legs separate from the ground, and in each of these stages, different kinetic variables intervene in the skill [5]. One of the most practical methods of kinetic analysis in jumping and landing skills is the use of a force-time curve and its analysis in two phases, eccentric and concentric, which can be applied to evaluate jumping and landing performance, neuromuscular, biomechanical and lower limb dynamics assessments [6–10].

The impulse parameter is vital in jumping and landing regarding the kinetic variables. From a biomechanical point of view, higher impulse magnitudes provoke more changes in pre-contact strategies during landing, affecting the body’s response to the ground contacts [11, 12]. When jumping on a force platform occurs, the main criterion is the force-time integral, i.e. the area below the force-time curve and the same impulse that provides valuable information for estimating the velocity-time profiles and position of the whole body mass center [6]. Optimal jump and landing performance is achieved through speed control, and according to Newton’s second law of motion, this speed is defined by the ratio of the generated impulse to the amount of momentum in the athlete [6]. In this law, a moment-impulse relationship is defined, during which the speed of the moment of separation from the ground can be determined. Previous studies suggest that jumping is performed in athletes of different disciplines, emphasizing the momentum variable, so more powerful vertical impulses create more vertical jumping heights [13, 14].

On the other hand, it has been proven that in addition to impulse, other kinetic variables derived from the force-time curve might influence jump and landing performances [12], such as success in related sports techniques, as well as created contact forces, trauma and collisions. Several investigations have reported the Rate of Force Development (RFD) as a valuable predictive index for adjusting temporal factors during the jumping performance. These findings suggest that RFD plays a significant role in modulating the timing and coordination of movement during jumping tasks. By considering the RFD, athletes and coaches can gain insights into optimizing the temporal aspects of their jumps, leading to improved performance outcomes. This highlights the practical relevance of RFD as a metric for assessing and refining the timing components of jumping movements in sports [15, 16]. For instance, previous studies provided evidence of a correlation between RFD and jumping performance [17, 18]. These findings lend support to the notion that RFD has a substantial impact on an individual’s ability to perform jumps effectively. The results of these studies highlight the importance of RFD in the context of jumping performance and suggest that improvements in RFD may lead to enhanced jumping abilities in athletes. So complete integrity is available while extracting kinetic information from this curve, and all these variables are interdependent in different phases. However, no distinct research has been found on the relationship between jump and landing variables in another stage as well as the relationship between impulse variables with each of these parameters, and most studies in this criterion focused on the amount of impulse produced during landing and its effect on jump performance [19–23].

Volleyball involves frequent and repetitive jumping and landing movements, which places substantial demands on the musculoskeletal system. Understanding the kinetics parameters during these actions can provide valuable insights into the biomechanical demands placed on the body during gameplay [24–26]. Moreover, previous research has indicated that improper jumping and landing techniques are associated with an increased risk of injury, particularly in sports such as volleyball, that involve high-impact movements. By studying the kinetics parameters of jumping and landing, the authors aim to identify potential risk factors or deficiencies in the technique that may contribute to injury occurrence. This knowledge can guide the development of injury prevention strategies and training interventions to improve safe and efficient movement patterns [9, 27–29]. Thus, the importance of impact on injury probability, optimizing jump and landing performance, and determining the loading factor of kinetic variables in a skill such as block jumping shows that the relationship between these variables should be investigated. The present study examined the relationships between impulse and kinetic variables during jumping and landing in elite young male volleyball players. Specifically, we aimed to identify the critical kinetic variables that predict impulse during landing and determine how different kinetic variables interact to affect impulse. By investigating these relationships, we have aimed to better understand the mechanisms underlying impulse production during jumping and landing, which could inform the development of effective training programs to enhance performance and reduce injury risk in volleyball players [30]. Overall, studying jumping-landing kinetics parameters in volleyball players can provide valuable information for injury prevention, performance enhancement, and the development of evidence-based training protocols specific to the sport. Building on previous research in this area [30, 31], we hypothesized that there would be significant correlations between various kinetic variables and impulse during jumping and landing in volleyball players.

Specifically, we hypothesized that variables related to concentric muscle actions, such as Peak Rate of Force Development concentric (PRFD _CON_) and Time to Peak Power Concentric (TPPC), would positively correlate with impulse at landing. The positive relationship between PRFD _CON_ and TPPC with landing impulses suggests that individuals who exhibit higher rates of force development and shorter times to peak propulsion during their jumps tend to generate greater landing impulses. This implies that athletes with superior power development capabilities and the ability to generate force quickly during the propulsive phase may experience higher impact forces upon landing [32–34]. We also hypothesized that variables related to eccentric muscle actions, such as eccentric RFD and Time to Peak Power Eccentric (TPPE), would positively correlate with impulse. We further hypothesized that PRFD _CON_ and TPPC would be the strongest predictors of impulse at the landing and that these variables would interact to affect impulse production. We tested these hypotheses to advance our understanding of the interplay between kinetic variables and impulse during jumping and landing in volleyball players. We tested these hypotheses to advance the aims of this study of the interplay between kinetic variables and impulse during jumping and landing in volleyball players.

## Materials and methods

### Participants

Eighteen young elite male volleyball players (age: 17.8 ± 0.9 years, height: 195.6 ± 3.1 cm, body mass: 77.3 ± 5.9 kg, body mass index: 21.2 ± 1.5 kg/m^2^) participated in this study voluntarily. Previous studies have consistently reported substantial correlations between force-time curve variables and jumping performance variables in volleyball players [30, 31, 35]. The G*Power software (University of Düsseldorf, Germany) was utilized for sample power calculations. To determine the required sample size power, an a priori correlation analysis using the point-biserial model was performed with the following information: α error probability (α err prob) = 0.05, power (1-β error probability) = 0.80, and effect size = 0.55. The analysis indicated that 16 participants would be needed to achieve 80.5% of the desired power. The subjects were a national team member who used to participate in regular volleyball training for ten sessions per week, each session for 2 h for two months of pre-season period. The convenience sampling method was used in the selection of the sample group. Participants used to be a member of the Premier League in the previous season of volleyball, and they declared no history of orthopaedic or neurological injuries in the past. Exclusion criteria for the study included individuals with any existing musculoskeletal or neurological condition that could influence the jumping and landing task. The present study was reviewed and approved in the laboratory of the National Olympic Committee and under the supervision of the National Volleyball Federation. Moreover, the Iran Research Ethics Committee of Sport Sciences Research Institute (No. IR.SSRI.REC.1151) confirmed all study processes and methods, which were performed by the ethical standards in the Helsinki Declaration. Additionally, before the study started, the participants and their parents were given detailed information about the project process and were asked to sign the informed consent form.

### Data collection

Measurements of the study were completed in the fourth week of the 8-week national training camp for the World Junior Championship. The rationale behind this approach was rooted in exercise science principles. This approach aimed to ensure that the subjects were physically prepared and capable of effectively executing the technical training phase. The study focused on the initial phase of the training protocol, which involves anatomical adaptation. At this stage, subjects were expected to have completed the necessary physical conditioning to prevent injuries and optimize their jumping and landing performance. A nutrition specialist monitored the players’ nutrition profile and physical fitness levels. Data collection was conducted in the Biomechanics Laboratory of the National Olympic Committee. Data from the players were collected between 5:00 and 7:00 p.m. during regular training hours. At the same time, the players had their lunch at 1:00 p.m. on the test day, and they consumed a 250-gr snack 30 min before starting the test procedure. Two experts conducted all the measurements in jumping and landing on one force platform (1000 Hz Kistler® -Switzerland).

**Fig. 1:**
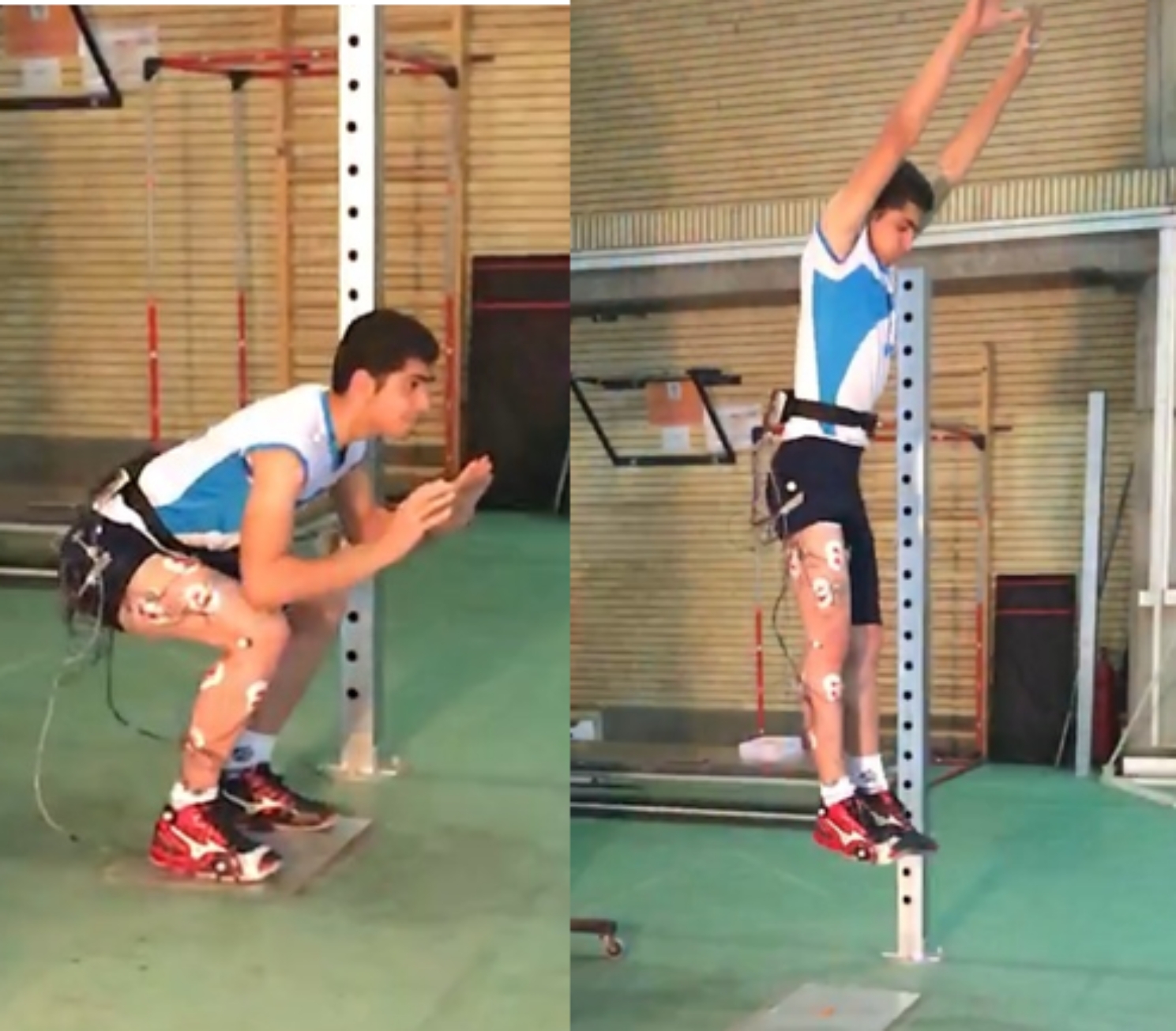
Block jump skill on force plate

The performed task in our study was volleyball block jump skill. The block jump is a specific type of jump used in volleyball, characterized by the absence of an arm swing. To execute a proper block jump involves a countermovement where the athlete initiates the jump with an eccentric contraction, followed by an amortization or braking phase, then rapidly extends the lower joints concentrically. The arm swing is minimized to maintain balance during the jump and landing. The hands must be positioned firmly to effectively block the attack of opponent spikers. They should be stretched wide to create a maximum surface area against the ball’s trajectory. During the landing phase after the block, it is recommended to land on both legs to reduce the risk of injury. The execution of the appropriate movement pattern is crucial in the block jump. It involves precise coordination of the lower body and the positioning of the hands to intercept the incoming ball effectively. The presence of the ball during the block jump can have two main effects: firstly, it can influence the timing of the jump, and secondly, it can affect the positioning of the hands. However, the mechanical performance of the lower joints is minimally affected by the presence of the ball. Hence, in our study, the evaluation of block performance did not involve the presence of a ball, whether stationary or during attackers’ hits. The rationale behind excluding the ball from the assessment was to concentrate solely on the block technique and its associated variables without considering the influence of ball trajectory or interaction. By isolating the block technique from ball-related factors, we aimed to investigate its specific components and performance indicators in volleyball, thereby enhancing our understanding of its mechanics and effectiveness. By eliminating the ball from the analysis, we aimed to isolate the factors directly related to the execution and effectiveness of the blocking action, allowing for a more targeted examination of the specific aspects under investigation [31, 36, 37] (Fig. 1).

To ensure standardized footwear conditions, participants were instructed to use new and reputable brands of shoes commonly used among volleyball players, including Mizuno®, Nike®, Adidas®, and Asics®. Shoe selection was based on their ability to provide optimal support, cushioning, and injury prevention during jumping and landing activities. Prior to the study, the condition of the shoes was carefully assessed to confirm their suitability, with particular attention given to signs of wear and tear.

The calibration process was conducted to gather data during the initial stage of the experiment. During this stage, the subjects maintained a stationary position for one second, after which the force plate was reset to zero. Following the calibration phase, the participants engaged in a standardized warm-up protocol lasting for 15 min, adhering to the routine volleyball training practices. After the warm-up period, a practice period was implemented before the actual data collection to familiarize each subject with the appropriate test procedure. Each subject was allowed to perform the test procedure multiple times, typically ranging from three to five practice attempts. After trials, to ensure the quality and consistency of the jumps, each participant performed three maximal jumps, and the best trial out of the three was selected for further analysis. This approach accounts for potential variations or performance fluctuations during a single jump. Since all participants were professional volleyball players, their landing technique was consistent and reflected the same quality they would use in their training and competitive settings.

Furthermore, each participant executed their landing strategy based on their routine, as visually assessed by the coaches during the test. The ground reaction force data were gathered directly according to the output of the Force Platform. The resultant of the 3D forces (Fx, Fy, Fz) for the time was the ground reaction force applied to the subjects at each time interval (related to the sampling frequency-1000 Hz). Force data were captured in both phases of jumping and landing, and further analysis was performed according to it. A fourth-order Butterworth filter with a cutting frequency of 50 Hz was used to filter the ground reaction force data. The Butterworth filter is an infinite impulse response filter commonly used for signal-processing tasks. The fourth order refers to the filter’s order, which indicates the complexity and effectiveness of the filtering process. Filtering the ground reaction force data using a fourth-order Butterworth filter with a cutting frequency of 50 Hz aimed to improve the signal-to-noise ratio and enhance the data quality. This filtering step likely helped to reduce unwanted high-frequency noise or interference, enabling a more accurate analysis, and interpretation of the ground reaction forces during the specified activity or experiment [2].

**Fig. 2:**
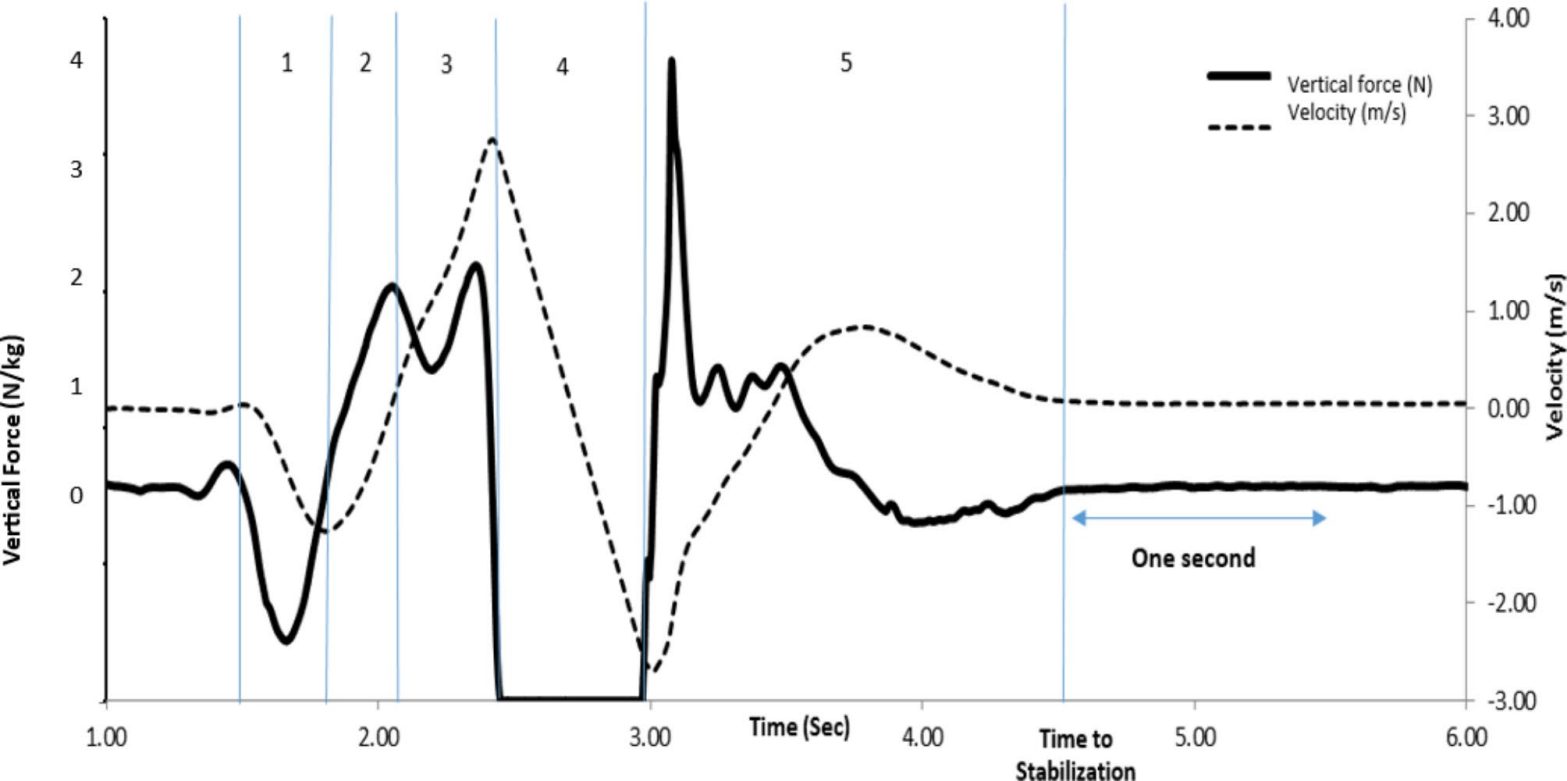
Graph of vertical reaction force, velocity and related temporal-phasic: (1) Initiation phase, (2) Eccentric phase (3) Concentric phase, (4) Jump phase (5) Landing phase to time to stability

The participants’ jump and landing kinetic data, including the variables listed below, were extracted directly from the force plate outputs, as shown in Fig. 2. First, the individual’s weight (mean force over 1 s when they stood still) was subtracted from the vertical force recorded by the force plate to calculate the velocity of the centre of mass. Then, the result was divided by the mass of each individual to determine the acceleration of the center of mass. Then, by integrating this variable, the velocity of the center of mass of the athlete was obtained (Fig. 2) [35, 38], after which the outputs listed below were analyzed by MATLAB (v9.14.0.2254940) software (Table 1). The jump height of each participant was estimated by Eq. 1: Jump height= (1/8) ×g×ft^2^ (Eq. 1). Equation 1 represents a mathematical formula for calculating the jump height, where “g” represents the acceleration due to gravity, and “ft” represents the time spent in the air during the jump.

**Table 1:** Descriptive measures of all variables in eccentric and concentric phases [30, 31]

Impulse (N.s):		The area below the force – time curve of landing from contact to stability.
**Time**	ET (ms)	Duration from the peak negative value to zero of the center of mass velocity.
	CT (ms)	Duration from the zero to the peak positive value of the center of mass velocity.
	TPPE (ms)	Time duration between the initiations till peak value of the peak power in eccentric phase.
	TPPC (ms)	Time duration between the initiations till peak value of the peak power in eccentric phase.
	IT (ms)	The period between the instant in which the velocity of the center of mass changes from zero to the peak negative value, this variable considered when the amount of ground reaction force on force plate became lower or higher than ± 10 N relative to body weight.
	AT (ms)	The duration between take off till contact with the force platform.
	TMLF (s)	Time duration between the contact instant and maximum vertical force.
	TMMLF (s)	Time duration between maximum and minimum vertical force till stability in landing phase.
	TS (s)	Time duration between contacts instant to the force platform till the magnitude of the vertical force stays stable within 5% of the jumper’s body weight.
**Force**	FECC _MAX_ (N/kg)	Maximum force calculated from eccentric phase normalized to body weight.
	FCON _MAX_ (N/kg)	Maximum force calculated from concentric phase normalized to body weight.
	F_maxL_ (N/kg)	The highest value of the force derived from force plate output in landing phase normalized to the body weight.
	F_minL_ (N/kg)	The lowest force peak derived from force plate output in landing phase normalized to the body weight.
	LR_max_ (N/kg/S)	The ratio of the maximum landing force normalized to the body weight to the time elapsed form contact to maximum force. Maximum to Minimum Loading Rate (LR _MAX-MIN_) (N/kg/s): Difference between maximum and minimum force of landing divided to interval time between two peaks.
**Power**	P Power _ECC_ (W/s)	Peak value of calculated power during eccentric phase normalized to body weight (Eq. 2): P = Fz VCOM.
	P Power _CON_ (W/s)	Peak value of calculated power during concentric phase normalized to body weight.
	A Power _ECC_ (W/kg)	Average of power during eccentric phase normalized to body weight.
	A Power _CON_ (W/kg)	Average of power calculated during concentric phase normalized to body weight.
**Rate of Force Development (RFD)**	PRFD _ECC_ (N/kg/s)	Peak value of the force ratio divided by elapsed time from the beginning of the eccentric phase until the peak force normalized to body mass.
	ARFD _ECC_ (N/kg/s)	Average of RFD calculated between each consecutive frame (0.001 s) in eccentric phase normalized to body mass.
	PRFD _CON_ (N/kg/s)	Peak value of the ratio of the force divided by elapsed time from the beginning of the concentric phase until the peak force normalized to body mass.
	ARFD _CON_ (N/kg/s)	Average of RFD was calculated between each consecutive frame according to Eq. 2 (P = F_z_ V_COM_) in concentric phase normalized to body mass.
	RFD _MAX_ECC_ (N/kg/s)	Maximum of eccentric force dividing to time elapsed between beginning of eccentric phase until the maximum force, normalized to body mass.
	RFD _MAX_CON_ (N/kg/s)	Calculated by dividing maximum force of concentric phase by time elapsed between beginning of concentric phase until maximum force normalized to body mass.

**Abbreviations**: ET, Eccentric Time; CT, Concentric Time; TPPE, Time to Peak Power Eccentric; TPPC, Time to Peak Power Concentric; IT, Initiation Time; AT, Airborne Time; TMLF, Time of Maximum to Minimum Landing Force; TMMLF, Time of Maximum to Minimum Landing Force; TS, Time to stability; FECC _MAX,_ Peak Power Eccentric; FCON _MAX_, Maximum Concentric Force; F_maxL_, Maximum Landing Force; F_minL_, Minumum Landing Force; LR_max_, Maximum Loading Rate; P Power _ECC,_ Peak Power Eccentric; P Power _CON_, Peak Power Concentric; A Power _ECC_, Average Power Eccentric; A Power _CON_, RFD, Rate of Force Development; Average Power Concentric; PRFD _ECC_, Peak RFD Eccentric; ARFD _ECC_, Average RFD Eccentric; PRFD _CON_, Peak RFD Concentric; ARFD _CON_, Average RFD Concentric; RFD _MAX_ECC_, RFD Maximum Eccentric; RFD _MAX_CON_, RFD Maximum Concentric

### Statistical analysis

The data analysis was conducted using SPSS version 26.0 (IBM, Armonk, NY, USA) software. Descriptive statistics, including the mean and standard deviation, were utilized to summarize and present the data. The dependent variable considered in this study was “impulse,“ while all the other kinetic and temporal variables were treated as independent variables. The Shapiro-Wilk test was used to determine whether the data showed a normal distribution and equality in variance. Subsequently, a Multiple Linear Regression Analysis was employed to examine the associations between impulse and kinetic variables during the jumping and landing phases. The significance level was considered as p ≤ 0.05.

## Results

The results of Shapiro-Wilk test showed that the data distribution was normal (p > 0.05). The results of descriptive statistics of the study variables are shown in Table 2.

**Table 2:** Descriptive data concerning variables under analysis

Variables	Mean ± SD	Variables		Mean ± SD	Variables	Mean ± SD
IP (N.S)	1579.80 ± 530.06	IT (ms)		361.88 ± 112.87	ET (ms)	286.00 ± 92.30
CT (ms)	344.17 ± 80.07	AT (ms)		585.88 ± 95.50	JP (cm)	40.85 ± 5.06
ARFD _ECC_ (N/kg/s)	38.44 ± 18.61	PRFD _ECC_ (N/kg/s)		6.80 ± 2.41	ARFD _CON_ (N/kg/s)	-27.16 ± 9.37
PRFD _CON_ (N/kg/s)	5.83 ± 2.01	FECC _MAX_ (W)		1.89 ± 0.25	FCON _MAX_ (W)	2.17 ± 0.16
P POWER_ECC_ (N.m/s)	-10.23 ± 4.31	P POWER _CON_ (N.m/s)		52.31 ± 13.68	TPPE (ms)	96.16 ± 54.88
TPPC (ms)	319.44 ± 55.98	A POWER _ECC_ (N.m/s)		-8.12 ± 3.17	A POWER _CON_ (N.m/s)	25.63 ± 6.68
RFD _MAX_ECC_ (N.m/s)	75.90 ± 38.07	RFD _MAX_CON_ (N.m/s)		76.64 ± 20.13	TS (s)	1.85 ± 0.93
F_maxL_ (W)	3.37 ± 0.88	TMLF (S)		0.085 ± 0.034	LR _max_ (BW/S)	46.91 ± 24.27
F_minL_ (W)	0.63 ± 0.13	TMMLF (S)		0.60 ± 0.15	LR _MAX−MIN_ (BW/S)	4.96 ± 2.25

**Abbreviations**: I, Initiation Phase; JP, Jumping Phase; ET, Eccentric Time; CT, Concentric Time; TPPE, Time to Peak Power Eccentric; TPPC, Time to Peak Power Concentric; IT, Initiation Time; AT, Airborne Time; TMLF, Time of Maximum to Minimum Landing Force; TMMLF, Time of Maximum to Minimum Landing Force; TS, Time to stability; FECC _MAX,_ Peak Power Eccentric; FCON _MAX_, Maximum Concentric Force; F_maxL_, Maximum Landing Force; F_minL_, Minumum Landing Force; LR_max_, Maximum Loading Rate; P Power _ECC,_ Peak Power Eccentric; P Power _CON_, Peak Power Concentric; A Power _ECC_, Average Power Eccentric; A Power _CON_, Average Power Concentric; RFD, Rate of Force Development; PRFD _ECC_, Peak RFD Eccentric; ARFD _ECC_, Average RFD Eccentric; PRFD _CON_, Peak RFD Concentric; ARFD _CON_, Average RFD Concentric; RFD _MAX_ECC_, RFD Maximum Eccentric; RFD _MAX_CON_, RFD Maximum Concentric

The outcomes of inferential statistics, and the results of the stepwise regression analysis can be observed in Table 3. It was found that there was a significant positive relationship between the PRFD _CON_, and impulse at the landing time (β = 0.537, p = 0.02). Moreover, it was observed that PRFD _CON_ (β = 0.497, p = 0.01) and the TPPC (β = 0.424, p = 0.04) were positively correlated with the impulse at the landing time. Finally, the analysis revealed that PRFD _CON_ and TPPC exhibited the highest factor loadings about the impulse.

**Table 3:** Results of stepwise regression analysis between impulse and other kinetic variables

Model		Unstandardized Coefficients		Unstandardized Coefficients	T	p
		**Beta**	**Std. Error**	**Beta**		
**1**	**Constant**	0.30	0.023		13.12	≤ 0.0001
	**PRFD**_**CON**_ **(N/kg/s)**	0.01	0.004	0.537	2.54	0.02
**2**	**(Constant)**	0.22	0.042		5.22	≤ 0.0001
	**PRFD**_**CON**_ **(N/kg/s)**	0.009	0.003	0.497	2.62	0.01
	**TPPC** **(ms)**	≤ 0.0001	≤ 0.0001	0.424	2.23	0.04

**Abbreviations**: RFD, Rate of Force Development; PRFD _CON,_ Peak RFD Concentric; TPPC, Time to Peak Power Concentric; the highlight indicates a significant result in the desired variable

## Discussion

The current study investigated the relationships between landing impulse and kinetic variables in a sample of junior elite male volleyball players. Our findings provide important insights into the factors influencing impulse production during jumping and landing and confirm the hypothesis that various kinetic variables are related to impulse in this population. Specifically, the present study reveals a significant positive relationship between the PRFD _CON_ and TPPC with impulse at landing. These two variables exhibited the highest factor loadings associated with impulse, indicating their crucial roles in determining jump performance and landing kinetics. These results highlight the significance of considering both concentric force development and the timing of peak power in optimizing impulse production during jumping and landing. The implications of our study extend to the biomechanics of jumping and landing in elite young male volleyball players. By elucidating the critical kinetic variables influencing impulse production, our research provides valuable new insights into the underlying performance mechanisms in this population.

Moreover, these findings have practical implications for training programs to enhance athletic performance and reduce the risk of injury. In light of our results, coaches and trainers can tailor their interventions to target the development of PRFD _CON_ and the optimization of TPPC, thereby improving athletes’ ability to generate higher impulses during jumps and landings. By integrating these findings into training protocols, athletes can strive for better performance outcomes while minimizing the potential for injuries associated with improper landing mechanics.

Impulse is one of the essential variables derived from the force-time curve [39], and it also has a decisive role in the quality of the jump and landing, as well as the height of the jump [40]. From a biomechanical point of view, the impulse variable covers a large part of the surface below the force-time curve, so it is predicted to significantly affect all the force and time variables derived from this curve or to be affected by them. For biomechanics researchers, one of the essential aspects of optimizing athletic performance and preventing injury is to determine the importance of the impact of each of these variables on impulse, but as far as we know, there is no study on this topic in the literature.

In our study, the variables derived from the force-time curve in all stages of jumping and landing were divided into five main phases: initiation, eccentric, concentric, jumping, and landing phases, each with separate force-time and dependent sub-variables. The present study examined extensive subsets of these variables to determine a more accurate relationship between kinetic parameters and landing impulse. In fact, by analyzing the reaction force of the earth and its subsets during landing, such as the kinetic and temporal variables produced by the force-time curve, it is possible to determine the vertical position of the body and the velocity of the body mass center [41]. Furthermore, according to the impulse-momentum relationship (Newton’s second law), the impulse of the reaction force on the athlete’s mass, which is the primary determinant of changes in the athlete’s speed during foot contact with the ground, can be accurately analyzed using these variables [19]. Therefore, the magnitude of the force and the moment applied to the joints of the body, as well as the impulse produced during landing (during foot contact with the ground), could potentially lead to various injuries in the lower extremities, especially when performing high-speed explosive skills [42].

In the present study, an interesting finding emerged from the analysis of the five phases of jump and landing. It was observed that the variables associated with the concentric phase exhibited higher factor loads compared to those of the other phases (as depicted in Fig. 2). The concentric phase occurs just before the separation phase from the ground. Therefore, the role of kinetic and kinematic functional variables in this phase can significantly impact the quality and biomechanics of jumping and landing skills following foot contact [36]. The jumping and landing skills evaluated in the present study was block jump, one of the most privileged and practical skills of volleyball, and it has an essential place in this sport. This skill, which involves the stretch-contraction cycle, is an explosive skill performed by flexing and extending the joints of the lower limbs- including the hip, knee, and ankle in a regular sequence and with specific timing and duration of eccentric and concentric phases. It includes that it is necessary to have the minor phase change time for its optimal implementation [37], the concentric phase and the pre-separation phase, which must enter the explosive phase of the body quickly [43]. Due to the explosive nature of the selected jump in the present study (block jump), the optimal execution depends on a sudden phase change from eccentric to concentric, requiring minor energy loss and the shortest time between these two phases to enter the jump phase. Therefore, athletes need to be able to transfer maximum energy and muscle strength to the lower limb long after the concentric phase to increase the height and optimal jump performance. Sports biomechanics experts attributed this situation to the conservation of energy stored in non-contractile components and the optimal use of contractile elements with the help of initial traction. Moreover, it should be noted that the concentric phase in the stretch-contraction cycle confirms positive work, and muscular muscle contraction may be associated with the production, and storage of high strength in the lower extremities.

The present study made a novel finding by identifying PRFD _CON_ as the primary factor influencing impulse. PRFD _CON_, as mentioned previously, represents the difference between maximum force and initial force during the concentric phase, divided by the difference in time to reach these forces. Our results align with previous studies, highlighting the crucial role of RFD, particularly in the concentric phase, in jump and landing performance [17, 44]. Since both the maximum force factor and the time-related RFD in the concentric phase have a special place in this variable, the impulse variable derived from both of these main force variables and the time and area below the related curve related to them. Therefore, a significant relationship between the peak RFD and impulse will probably be justified. In addition, perhaps due to the importance of the concentric phase, the RFD in this phase also has the most significant effect on the impulse during landing.

Another critical factor identified in the present study is the TPPC. This variable, along with other factors such as power and peak power, reflects specific physical fitness characteristics in athletes and plays a significant role in jump and landing performance [45]. For instance, Lorenz et al. [46] showed that peak power output could be the most crucial jump and landing performance predictor. Also, another study emphasized the peak power output’s effect on jump height and landing quality [31, 35]. Power is the ability of skeletal muscles to produce force quickly and is the product of the amount of force multiplied by the velocity of contraction. Therefore, having high power can positively affect the athlete’s type of landing, that is, the contact with the ground. Lastly, the effect and high factor load time of reaching this variable in the critical concentric phase on the impulse can be significant in future studies.

Our study offers novel contributions to understanding jumping and landing biomechanics in elite young male volleyball players. By identifying and emphasizing the critical kinetic variables related to impulse production, our research is valuable for optimizing training strategies, enhancing performance, and promoting injury prevention in this athletic population. In our study, it is essential to acknowledge some limitations. One major limitation of this study is the relatively small sample size, which may limit the generalizability of our findings to other populations. Future studies with larger and more diverse samples would be needed to confirm the generalizability of our results. Another potential limitation of this study is that we did not track our participants’ nutrition and hydration status during the study period. This could have affected their performance and recovery and confounded our results. Future studies should consider incorporating measures of nutritional and hydration status to understand better these factors’ role in jump performance and landing kinetics.

To extend the findings of this study, future research could explore additional biomechanical markers in male and female athletes across different sports. For example, it would be interesting to investigate the relationship between impulse and kinetic variables during jumping and landing in other sports that require explosive movements, such as basketball, track and field, and gymnastics. By expanding the scope of our research, we can gain a more comprehensive understanding of the underlying mechanisms of athletic performance and help inform the development of more effective training programs.

## Conclusion

Our study emphasizes the crucial role of specific kinetic variables, such as PRFD _CON_ and TPPC, in determining impulse during the landing phase of jumping. By closely considering these variables, coaches can create targeted training programs to enhance performance and minimize the risk of injury. When developing exercises and teaching proper techniques for explosive skills like block jumping, it is vital to account for the connections between different kinetic variables and their impact on impulse during landing. By prioritizing the development of critical parameters such as PRFD _CON_ and TPPC, coaches can assist athletes in achieving more effective and efficient jumps while minimizing injury risks. Furthermore, it may be advantageous to break down the various phases of jumping and landing skills and focus on specific aspects of technique and form that contribute to improved impulse production. Overall, our findings hold significant implications for athletic training and performance by emphasizing the importance of comprehending the intricate interplay between different kinetic variables during jumping and landing. Coaches can apply these insights to help athletes optimize their performance, reduce the risk of injury, and develop more effective and efficient techniques for explosive movements.

## Data Availability

The datasets used and/or analyzed during our study are available from the corresponding author upon reasonable request, provided the appropriate permits are obtained from the relevant authorities.

## References

[1] Daneshjoo A, Nobari H, Kalantari A (2021). Comparison of knee and hip kinematics during landing and cutting between Elite Male Football and Futsal Players. Healthcare.

[2] Zahradnik D, Uchytil J, Farana R (2014). Ground reaction force and Valgus knee loading during landing after a Block in Female Volleyball Players. J Hum Kinet.

[3] Hoseini A, Zarei M, Nobari H (2022). Isokinetic muscle strength cannot be related to the odds ratio of musculoskeletal injuries in young elite wrestlers. BMC Sports Sci Med Rehabil.

[4] Sinsurin K, Vachalathiti R, Jalayondeja W (2013). Different sagittal angles and moments of lower extremity joints during single-leg Jump Landing among various directions in basketball and volleyball athletes. J Phys Ther Sci.

[5] Davis DS, Briscoe DA, Markowski CT (2003). Physical characteristics that predict vertical jump performance in recreational male athletes. Phys Ther Sport.

[6] Ruddock AD, Winter EM (2016). Jumping depends on impulse not power. J Sports Sci.

[7] Xu D, Quan W, Zhou H (2022). Explaining the differences of gait patterns between high and low-mileage runners with machine learning. Sci Rep.

[8] Ahmadi M, Nobari H, Ramirez-Campillo R (2021). Effects of Plyometric Jump Training in sand or rigid surface on Jump-Related biomechanical variables and physical fitness in female Volleyball players. Int J Environ Res Public Health.

[9] Xu D, Jiang X, Cen X (2020). Single-Leg Landings following a Volleyball Spike May increase the risk of Anterior Cruciate Ligament Injury more than landing on both-legs. Appl Sci.

[10] Xu D, Lu Z, Shen S (2021). The differences in lower extremity joints Energy Dissipation Strategy during landing between athletes with symptomatic patellar tendinopathy (PT) and without Patellar Tendinopathy (UPT). Mol Cell Biomech.

[11] Harry JR, Freedman Silvernail J, Mercer JA (2017). Comparison of pre-contact joint kinematics and vertical impulse between vertical jump landings and step-off landings from equal heights. Hum Mov Sci.

[12] Mathiyakom W, McNitt-Gray JL, Wilcox R (2006). Lower extremity control and dynamics during backward angular impulse generation in forward translating tasks. J Biomech.

[13] *Med & Sci Sport & Exerc* 1996; 28: 1402–1412. 10.1097/00005768-199611000-00009 8933491

[14] Hara M, Shibayama A, Takeshita D (2008). A comparison of the mechanical effect of arm swing and countermovement on the lower extremities in vertical jumping. Hum Mov Sci.

[15] Cronin J, Sleivert G (2005). Challenges in understanding the influence of maximal power training on improving athletic performance. Sport Med.

[16] Haff GG, Carlock JM, Hartman MJ (2005). Force–time curve characteristics of dynamic and isometric muscle actions of Elite Women Olympic Weightlifters. J Strength Cond Res.

[17] Laffaye G, Wagner PP, Tombleson TIL (2014). Countermovement Jump Height. J Strength Cond Res.

[18] McLellan CP, Lovell DI, Gass GC (2011). The role of rate of Force Development on Vertical Jump performance. J Strength Cond Res.

[19] Kawamori N, Nosaka K, Newton RU (2013). Relationships between Ground reaction impulse and Sprint Acceleration performance in Team Sport athletes. J Strength Cond Res.

[20] Yamashita D, Murata M, Inaba Y (2020). Effect of Landing posture on Jump Height calculated from Flight Time. Appl Sci.

[21] McNitt-Gray J. Impulse generation during jumping and landing movements. ISBS-Conference Proceedings Archive., pp. 95–99.

[22] Slamka M, Lesko M, Psalman V. Measurement of amortisation and take off force impulse during the jump with both legs. ISBS-Conference Proceedings Archive., pp. 319–322.

[23] Suchomel TJ, McKeever SM, Sijuwade O (2023). Propulsion phase characteristics of Loaded Jump Variations in resistance-trained women. Sports.

[24] Garcia S, Delattre N, Berton E (2022). Comparison of landing kinematics and kinetics between experienced and novice volleyball players during block and spike jumps. BMC Sports Sci Med Rehabil.

[25] Tilp M. The biomechanics of volleyball. In: *Handbook of Sports Medicine and Science*. Chichester, UK: John Wiley & Sons, Ltd, pp. 29–37.

[26] Daneshjoo A, Nobari H, Kalantari A (2021). Comparison of knee and hip kinematics during landing and cutting between Elite Male Football and Futsal Players. Healthc (Basel).

[27] Tillman MD, Hass CJ, Brunt D (2004). Jumping and landing techniques in Elite Women’s Volleyball. J Sports Sci Med.

[28] Powers CM (2010). The influence of abnormal hip mechanics on knee Injury: a biomechanical perspective. J Orthop Sport Phys Ther.

[29] Peña J, Gil-Puga B, Piedra A, Altarriba-Bartés A, Loscos-Fàbregas E, Chulvi-Medrano I, Casals M (2023). García de Alcaraz A. Epidemiology and risk factors in young female athletes: basketball, soccer, and volleyball. Apunt Educ Física y Deport.

[30] Fatahi A, Yousefian Molla R, Ghomsheh T, Ameli M (2021). Relationship between temporal variables and rate of force development during block jump skill in junior volleyball players. J Adv Sport Technol.

[31] Sarvestan J, Cheraghi M, Sebyani M (2018). Relationships between force-time curve variables and jump height during countermovement jumps in young elite volleyball players. Acta Gymnica.

[32] Hornsby W, Gentles J, MacDonald C (2017). Maximum strength, rate of Force Development, Jump Height, and peak power alterations in weightlifters across five months of training. Sports.

[33] Barker LA, Harry JR, Mercer JA (2018). Relationships between Countermovement Jump Ground reaction forces and Jump Height, reactive Strength Index, and Jump Time. J Strength Cond Res.

[34] Zemková E, Poór O, Pecho J (2019). Peak rate of Force Development and Isometric Maximum Strength of back muscles are Associated with Power Performance during load-lifting tasks. Am J Mens Health.

[35] Sarvestan J, Svoboda Z, de Oliveira Claudino JG (2020). Force-time curve variables of countermovement jump as predictors of volleyball spike jump height. Ger J Exerc Sport Res.

[36] Fatahi A, Sadeghi H, Yousefian Molla R (2019). Selected kinematic characteristics analysis of knee and ankle joints during Block Jump among Elite Junior Volleyball Players. Phys Treat Specif Phys Ther J.

[37] Fatahi A, Yousefian Molla R, Ameli M (2020). Three-dimensional analysis of selected kinetics and impulse variables between Middle and Wing Volleyball Attackers during Block Jump based on Integration Method. J Adv Sport Technol.

[38] Robertson DGE, Caldwell GE, Hamill J, Kamen G, Whittlesey S (2013). Research methods in biomechanics.

[39] Mizuguchi S, Sands WA, Wassinger CA (2015). A new approach to determining net impulse and identification of its characteristics in countermovement jumping: reliability and validity. Sport Biomech.

[40] Linthorne NP (2001). Analysis of standing vertical jumps using a force platform. Am J Phys.

[41] Cheuvront SN, Kenefick RW, Ely BR (2010). Hypohydration reduces vertical ground reaction impulse but not jump height. Eur J Appl Physiol.

[42] McNitt-Gray JL (1991). Kinematics and impulse characteristics of Drop Landings from three Heights. Int J Sport Biomech.

[43] Fatahi A, Molla Y, Ameli M (2020). Comparative analysis of jumping and landing velocity of the Young Elite Spikers of the Iranian National Volleyball Team while performing Block Jump. J Sport Biomech.

[44] Kawamori N, Rossi SJ, Justice BD (2006). Peak force and rate of Force Development during Isometric and dynamic Mid-Thigh Clean pulls performed at various intensities. J Strength Cond Res.

[45] Kirby TJ, McBride JM, Haines TL (2011). Relative net Vertical Impulse determines jumping performance. J Appl Biomech.

[46] Brini S, Boullosa D, Calleja-González J (2022). Impact of combined versus single-mode training programs based on drop jump and specific multidirectional repeated sprint on bio-motor ability adaptations: a parallel study design in professional basketball players. BMC Sports Sci Med Rehabil.

